# Sensorimotor accuracy and dynamic muscular endurance in war veterans (amateur athletes) during rehabilitation after battle trauma

**DOI:** 10.1192/j.eurpsy.2023.2192

**Published:** 2023-07-19

**Authors:** S. Fedorchuk, I. Pryma, I. Kohut, N. Krushynska, S. Tukaiev

**Affiliations:** 1 National University of Ukraine on Physical Education and Sport, Kyiv, Ukraine; 2 Università della Svizzera italiana, Faculty of Communication, Culture, and Society, Institute of Public Health, Lugano, Switzerland; 3 National Taras Shevchenko University of Kyiv, Kyiv, Ukraine

## Abstract

**Introduction:**

During the war, the rehabilitation of combatants after injuries of varying degrees of complexity has particular importance. The effectiveness of rehabilitation of athletes-war veterans can be assessed by the level of physical performance, functional properties and state of all body systems. The reduction of functional asymmetry, accuracy of the sensorimotor response and dynamic muscular endurance (DME) of hand movement by the tapping test are effective and objective indicators of physical rehabilitation.

**Objectives:**

The aim of this study was to evaluate functional asymmetry, accuracy of sensorimotor response and dynamic muscular endurance of hand movement by tapping test during the rehabilitation of amateur field-and-track athletes after battle traumas.

**Methods:**

10 war veterans (amateur field-and-track athletes, right-handed male aged between 28 and 60 years) took part in the study. To determine the state of psychophysiological functions and the maximum tempo of movement of the hand Diagnostic complex “Diagnost-1” (Ukraine) was used. We analyzed indicators of the tapping test, indicators of a simple visual-motor reaction (SVMR) and a reaction of choosing one of three signals (RCh1-3) separately for the right and left hand, indicators of a reaction of choosing two of three signals (RCh2-3). Non-parametric statistics methods (Spearman’s rank correlation coefficient) were used to process data.

**Results:**

The accuracy of the sensorimotor reaction (according to the SVMR indicators) corresponded to the average level in the majority of athletes (60%). The indicators of the simple response of the choice of RCh1-3 were below the average (20%) or at a reduced level (50% of the examined). DME of the dominant hand was high or medium in 40% and 60% participants respectively. In 90% of the surveyed athletes, asymmetry in terms of the tapping test is moderately expressed. Results showed the significant correlation between the stability index of the simple visuomotor reaction (SVMR) and the dynamic muscular endurance (DME) indicators for the dominant and subdominant hand (r = 0.75, r = 0.71, p<0.05, respectively) - the greater DME corresponded to the lower stability of the SVMR. Latency periods of simple visuomotor reaction and their components (motor components of SVMR, RCh1-3, RCh2-3 reactions and the time of central processing of information in choice reactions) were not associated with tapping test indicators.

**Conclusions:**

Therefore, it can be assumed that motor components of a simple visuomotor reaction, choice reactions and the time of central processing of information in choice reactions were not related to dynamic muscle endurance. The revealed interrelations between the stability of the sensorimotor response and the dynamic muscular endurance of the hand movement can be indicators of the successful rehabilitation of amateur athletes after injury.

**Disclosure of Interest:**

None Declared

